# Lessons in Gynæcology

**Published:** 1879-08

**Authors:** 


LESSONS IN GYNÆCOLOGY. By Wm. Gooddell, A.M., M.D., Physician to the Preston Retreat; Prof. of Clinical Gynæcology in the University of Pennsylvania, etc., etc.; with eighty illustrations. D. G. Brinton, publisher, Philadelphia. 1879.

We have received through Messrs. J. J. & S. P. Richards a copy of this work of three hundred and seventy odd pages.

The arrangement of the subject matter consists in a series of lessons comprising twenty-nine lectures, and treating very perpicuously of the various uterine troubles and their complications.

In lesson II. we find some important suggestions in the way of diagnosis and treatment of urethral affections in the female. Caruncle of this part we have found very troublesome, and find much benefit from his remarks under this head.

The lessons devoted to the surgical operations on the perineum, vagina and cervix for the various diseases affecting those parts are comprehensive and instructive. The subjects of displacements and adventitious growths of the uterus, diseases of the ovaries and the relation of neurasthenia to diseases of the womb, are treated of to the fullest extent, and add much to the value of the book. The last three lessons are devoted to hygenic considerations, or the causes and prevention of female diseases. In this connection is mentioned the influence that faulty closets bear in the promotion of diseases of women, and the remarks under these heads are good and to the point.

